# Supplementary material for: Synthesis and Intramolecular Energy- and Electron-Transfer of 3D-Conformeric Tris(fluorenyl-[60]fullerenylfluorene) Derivatives
Source: Molecules. 2019 Sep 13;24(18):3337. doi: 10.3390/molecules24183337 (PMC6766839; doi:10.3390/molecules24183337)
Supplement: Supplementary file 1 [file molecules-24-03337-s001.zip › molecules-577185-SI.pdf]

Synthesis and Intramolecular Energy- and Electron-Transfer of 3D-Conformeric Tris(fluorenyl-[60]fullerenylfluorene) Derivatives

He Yin [1], Min Wang [1], Loon-Seng Tan [2], and Long Y. Chiang [1,*]

1 Department of Chemistry, University of Massachusetts Lowell, Lowell, Massachusetts 01854, United States

3 Functional Materials Division, AFRL/RXA, Air Force Research Laboratory, Wright-Patterson Air Force Base, Dayton, Ohio 45433, United States

* Author to whom correspondence should be addressed: E-Mail: Long_Chiang@uml.edu; Tel.: +1-978-934-3663; Fax: +1-978-934-3013

Supporting Information

The spectroscopic characterization of tris[(DPAF-C_{2M})-C_{60}(>DPAF-C_9)] (**4**-C_{2M-9}) was based on the structural extension of the previous generation of tris[C_{60}(>DPAF-C_9)] with an extensive characterization [S1]. Spectroscopic characterizations of tris[C_{60}(>DPAF-C_9)] was built based on its precursor 3D-conformer tris(DPAF-C_9) with both ^{1}H and ^{13}C NMR characterizations (Figure S1) [S2]. Two 3D-conformers of tris(DPAF-C_9) as a *cis-cup* form and *trans-chair* form were separated and purified via chromatographic TLC and HPLC techniques. Yield optimization of the *cis-cup* form was guided by the molecular simulation of tris(DPAF-C_9) with the optimization at the B97-D3/SVP level of the density functional theory (DFT) calculations [S2,S3]. Therefore, the intermediate precursor of the *cis-cup* form was applied directly in the current manuscript by citing our previous publications. These data of Figures S1 and S2 are placed in the supporting information.

Attachment of C_{60}(>DPAF-C_9) to the *cis-cup* form intermediate followed the similar procedure used in the preparation of tris[C_{60}(>DPAF-C_9)] (Figure S2). Accordingly, the yield and spectroscopic characterization of tris[(DPAF-C_{2M})-C_{60}(>DPAF-C_9)] (**4**-C_{2M-9}) were based on the data reference of tris[C_{60}(>DPAF-C_9)] in comparison.

Regarding to the Friedel-Crafts acylation at the C7 of the fluorene ring, the high regio-selectivity was proven by the x-ray single crystal structural characterization of C_{60}(>DPAF-C_2) reported previously [S4,S5]. Similar reaction conditions were applied to produce the same regio-selectivity followed by ^{1}H NMR verification using the spectroscopic data of C_{60}(>DPAF-C_2) as a base for comparison. Accordingly, the assignment of three fluorene ring protons next to the keto group, as reported [S5], was used for comparison in the same proton peak assignments in Figure S3.

The yield of Friedel-Crafts acylation product tris(BrDPAF-C_{2M}) in this manuscript was 48%, as described in the experimental section, that can be compared with 53% reported for tris(BrDPAF-C_9) [S1], 67% [S5] and 68% [S6] reported for BrDPAF-C_9 (single addition), and 66% for BrDPAF-C_2 (single addition) [S5].

For the structural correlation, three protons (H_a, H_b, H_c) of the precursor compound BrDPAF-C_9 for C_{60}(>DPAF-C_9) with the corresponding chemical shift at δ 7.63 (doublet, H_a), 7.94 (doublet, H_b), and 7.90

(singlet, H_c), respectively [S5], in Figure S3a [S6] were applied as the reference. By comparing those of BrDPAF-C_9 (Figure S3a) and tris(BrDPAF-C_{2M}) (Figure S3b), the chemical shift and integration of these protons centered at δ7.64 (H_a) and 7.98 (H_b and H_c) was found to be in the similar range. They can also be correlated to those of BrDPAF-C_{2M} (Figure S3c) showing the corresponding sharp proton peaks at δ7.67 (doublet, H_a), 7.99 (doublet, H_b), and 8.0 (singlet, H_c). A close resemblance of these proton peak characteristics revealed the same type of Friedel-Crafts reaction occurring at C7 of the fluorene ring. Furthermore, apparent down-fielded shift of chemical shift values to δ7.83 (H_a), 8.48 (H_b), and 8.34 (H_c) was detected upon the attachment of a C_{60} cage to form C_{60}(>DPAF-C_9). These chemical shifts of proton peaks were also used to assist the proton assignment of the product tris[(DPAF-C_{2M})-C_{60}(>DPAF-C_9)] (**4**-C_{2M-9}) (Figure 2).

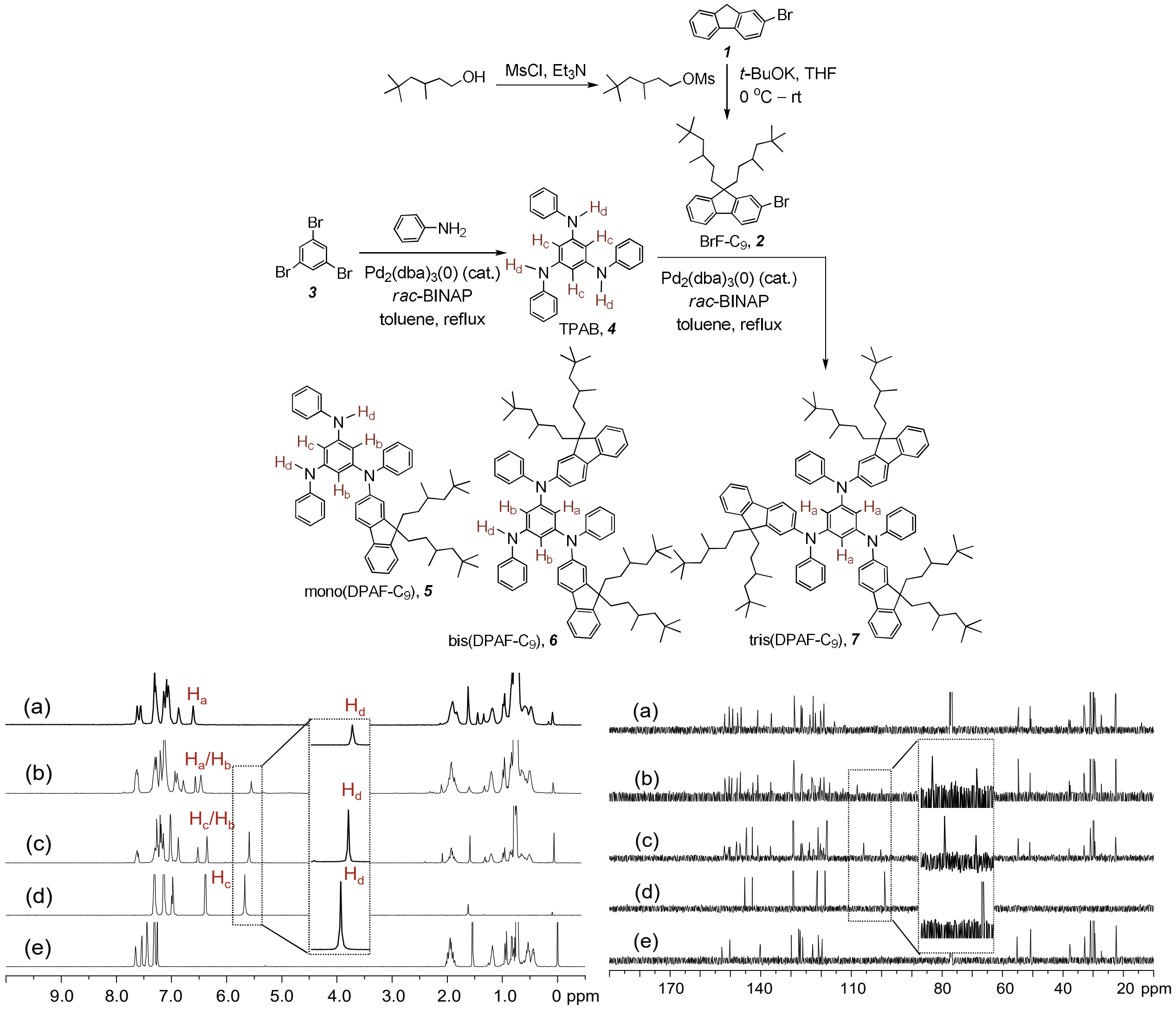

^{1}H NMR spectra of (a) tris(DPAF-C_9) **7**, (b) bis(DPAF-C_9) **6**, (c) mono(DPAF-C_9) **5**, (d) TPAB **4**, and (e) BrF-C_9 **2**.

^{13}C NMR spectra of (a) tris(DPAF-C_9) **7**, (b) bis(DPAF-C_9) **6**, (c) mono(DPAF-C_9) **5**, (d) TPAB **4**, and (e) BrF-C_9 **2**.

Figure S1. Synthetic route to the preparation of tris(DPAF-C_9) with reaction reagents provided and its ^{1}H and ^{13}C NMR spectroscopic characterizations [S2].

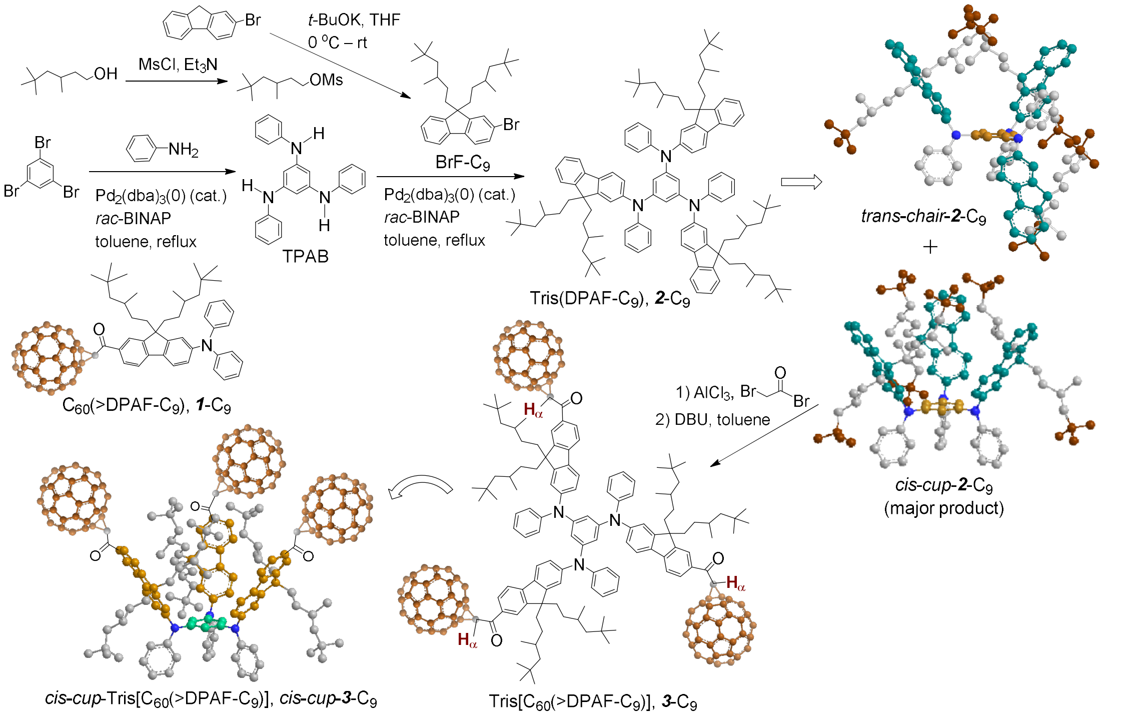

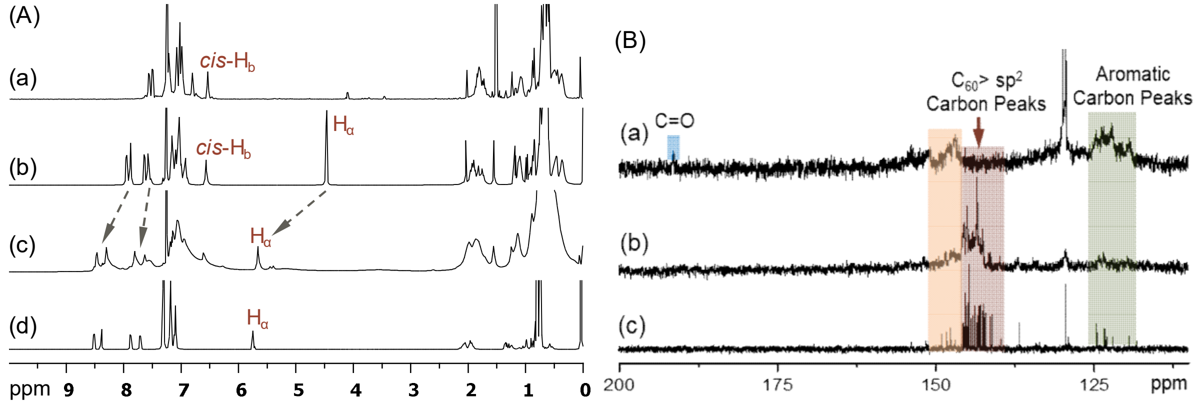

Figure S2. (A) ^{1}H NMR spectra ($CDCl_3$) of (a) *cis-cup*-tris(DPAF-C_9) (*cis-cup*-**2**-C_9), (b) *cis-cup*-tris(BrDPAF-C_9), (c) *cis-cup*-tris[C_{60}(>DPAF-C_9)] (*cis-cup*-**3**-C_9), and (d) C_{60}(>DPAF-C_9) as a reference for comparison. (B) ^{13}C NMR spectra of (a) *cis-cup*-tris(BrDPAF-C_9), (b) *cis-cup*-tris[C_{60}(>DPAF-C_9)], and (c) C_{60}(>DPAF-C_9) (**1**-C_9), showing in (b) the main group of fullerenyl sp^2 carbon peaks at δ140–147 that indicated the attachment of C_{60}> cages on **2**-C_9, as compared with those of (Bc) [S1].

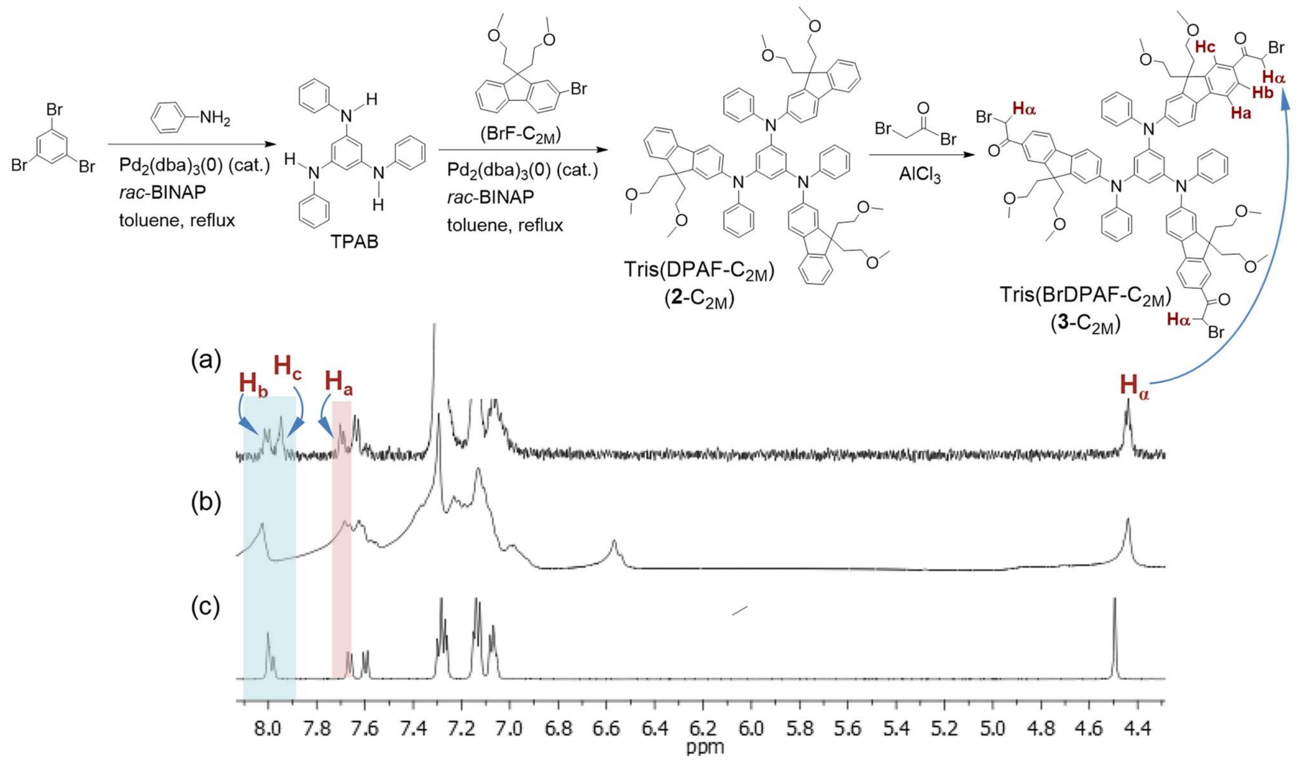

Figure S3. Partial synthetic routes for the preparation of tris(BrDPAF-C_{2M}) (**3**-C_{2M}), as a precursor for tris[(DPAF-C_{2M})-C_{60}(>DPAF-C_9)] (**4**-C_{2M-9}), with four protons labeling in **3**-C_{2M}. ^{1}H NMR spectra of (a) BrDPAF-C_9, (b) tris(BrDPAF-C_{2M}), and (c) BrDPAF-C_{2M} in $CDCl_3$.for comparison.

As stated above, the 3D-configuration of **4**-C_{2M-9} was determined at the stage of its precursor intermediate, tris(DPAF-C_{2M}) (**2**-C_{2M}). The isolation and purification of this compound via chromatography ($R_f = 0.6$, hexane–ethylacetate (1:1, v/v) as the eluent, as indicated in the experimental section) followed those of tris(DPAF-C_9) described in Figure S4 [S3].

To understand the possible 3D-configurations of tris(DPAF-C_9) and tris(DPAF-C_{2M}), we carried out the molecular simulation and calculations of the heat of formation (ΔH_f), via the semi-empirical PM3 method (Spartan 08) and B3LYP/6-31G* level of theory [S2]. Independent calculations were also performed with the optimization at the B97-D3/SVP level of the density functional theory (DFT) [S3]. These included several similar derivatives of tris(DPAF-C_n) [n = 2 (ethyl), 2M ($MeOC_2H_4$, **2**-C_{2M}). 6 (*n*-hexyl), 7 (*n*-heptyl), 8 (*n*-octyl), and 9 (**2**-C_9)] investigated in the calculations. Three possible 3D-conformers were examined to find that the propeller form led to the highest conformation energy with the *chair* and *cup* geometries being the lower energy conformers.

Surprisingly, it revealed unexpectedly high stability of the *cup*-form with other forms in an order of *chair* ≈ *cup* > *propeller* for C_9 substituents (–9.19, –9.13, and –4.40 kcal/mol, respectively) and the highest stability of *cup*-forms (*cup* > *chair* > *propeller*) for the slightly less crowded *n*-C_6, C_7, and C_8 substituents. Such *cup*-form formation tends to be more preferable as the alkyl chains at 9-fluorenyl position become longer as can be seen in the differential heat of formation $\Delta\Delta H_f$ for both *cup–propeller* and *cup–chair* energy gaps. These results

imply that hydrophobic interactions between three alkyl chains as well as CH/π interaction between the alkyl group and the fluorenyl ring plays an important role for the preferential formation of the *cup*-form. The DFT calculation at B3LYP/6-31G* level of theory for some alkyl groups resulted in the similar tendency, although the values suggested the slightly more stable *chair*-form even for *n*-C_6 group as compared with the *cup*-form, probably due to the lack of consideration on solvent polarity which affects to hydrophobic interactions (Table S1).

Table S1. DFT calculation of heat of formation for tris(DPAF-C_n).[a]

R[b]	ΔH_f (kcal/mol)		$\Delta\Delta H_f$ (kcal/mol)
(C_n)	*cis* (*cup*)	*trans* (*chair*)	*cup–chair*
Et	–1922585.7	–1922609.1	23.4
$MeOC_2H_4$	–2353750.4	–2353762.0	11.6
n-C_6	–2514673.7	–2514677.3	3.6
C_9	–2958697.2	–2958703.8	6.6

[a] Calculated at B3LYP/6-31G* level of theory using SPARTAN08.
[b] Substituents at 9-fluorenyl position.

The unexpected stability of *cup*-form may be explained by the torsional angle of the center benzene ring defined by the position of aryl groups on nitrogen atoms. The calculated torsional (dihedral) angles between the aryl–nitrogen C–N bond and the center benzene ring C–C bond for two *cis*-conformers of tris(DPAF-C_9) (*cup*-form and *propeller*-form) obtained from the optimized 3D-structure. The angle strain of the *cup*-form was found to be much less than that of the *propeller*-form. The angle strain of *trans*-conformers is somewhat higher than that of the symmetrical *cup*-form due to unavoidable unsymmetrical conformation of the *chair*-form. Therefore, the highly symmetrical *cis*-*cup*-isomer of tris(DPAF-C_9) can be formed exclusively.

Between the *chair* and *cup* geometries, the tris-*chair* form is predicted to be more stable by $\Delta\Delta G$ = 2.1 kcal/mol. However, there is an entropic preference for the *cis*-*cup* form amounting to ~3 kcal/mol (compare $\Delta\Delta H_{gas}$ and $\Delta\Delta G_{gas}$). Furthermore, the tris-*cup* form is predicted to have a stronger dipole [dipole(chair) = 2 D; dipole(cup) = 3.6 D], by virtue of possessing more hydrophilic and lipophilic ends than the tris-*chair* form, and should thus increase in preference in more polar media. As expected, with the inclusion of chloroform solvation, the preference for the tris-*chair* form is reduced to nearly zero, $\Delta\Delta G$ = 0.1 kcal/mol. Nonetheless, the possibility that the cage-like C_3 symmetric tris-*cup* form could be present in appreciable quantity is intriguing based on its unique structural symmetry.

Experimentally, we found that the *cis*-*cup* form [R_f = 0.65–0.7, SiO_x, hexane–ethylacetate (9.5:0.5, v/v) as the eluent] led to a sharp singlet proton peak at δ 6.55 (Figure S4e) that was assigned for three phenyl H_a protons located at the central benzene core of tris(DPAF-C_9). Whereas the spectrum (Figure S4d) of tris-*chair* conformer isolated at TLC R_f = 0.6–0.65 (*trans*-**1**-C_9 of Figure S4d with two fluorene rings facing upward and one fluorene ring facing downward forming a configuration of chair-like form) displayed two proton peaks at the same chemical shift region in an integration ratio of 2:1. Therefore, we assigned the peaks at δ 6.55 and 6.48 for the

chemical shift of central phenyl protons $H_{a'}$ and $H_{b'}$, respectively. These two spectra provided crucial information to allow us making differentiation on the corresponding 3D-conformation of *cis*-*cup*-**1**-C_9 and *trans*-*chair*-**1**-C_9 of Figure S4 [S3].

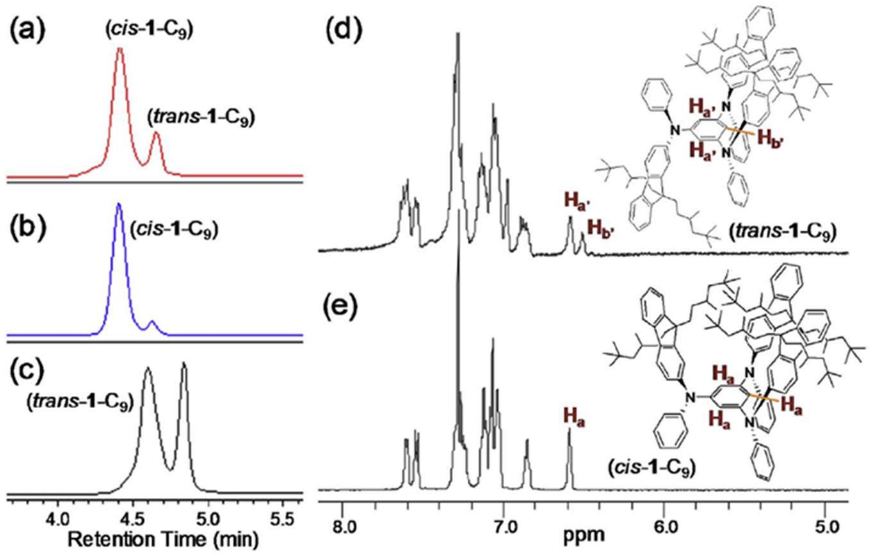

Figure S4. HPLC elution of (a) a crude TLC band indicating a roughly 7:3 area ratio of the tris-*cup* conformer tris(DPAF-C_9) (*cis*-**1**-C_9) vs the tris-*chair* 3D conformer *trans*-**1**-C_9, (b) a narrow topcut TLC band of *cis*-**1**-C_9, and (c) a narrow bottom-cut TLC band of *trans*-**1**-C_9 and ^{1}H NMR spectra ($CDCl_3$) of (d) trischair trans-**1**-C_9, (e) tris-*cis cup*-**1**-C_9 [S3].

In the case potential formation of regio-isomers of **4**-$C_{2M\text{-}9}$ at the <C60> moiety, since the monoadduct structure of C_{60}(>DPAF-C_9) is well-defined, with the assistance of x-ray single crystal structural analysis of C_{60}(>DPAF-C_2) [S4,S5], its attachment on tris(BrDPAF-C_{2M}) (**3**-C_{2M}) was believed to be governed by the bulkiness and steric hindrance of both relatively large entities to result in only a limit number of region-isomers on the C_{60} cage. This was proven by the ^{1}H NMR spectrum of **4**-$C_{2M\text{-}9}$ showing only several H_α and H_α' proton peaks at roughly δ 5.2–5.8 (Figure 2c) instead of a broad band normally for the existence of a large number of region-isomers. To our surprise, a peak at 524 cm^{-1} assigned for the characteristic Infrared absorption band of a half-C_{60} cage was detected (Figure S5d), resembling those of the monoadduct C_{60}(>DPAF-C_9) (**1**-C_9, Figure S5b) and tris[C_{60}(>DPAF-C_9)] (Figure S5c) at the identical 524 cm^{-1}. It implied the structure of the major regio-isomer products having both addend moieties located at the same half sphere of a C_{60} cage that leaves the other half sphere of C_{60} untouched.

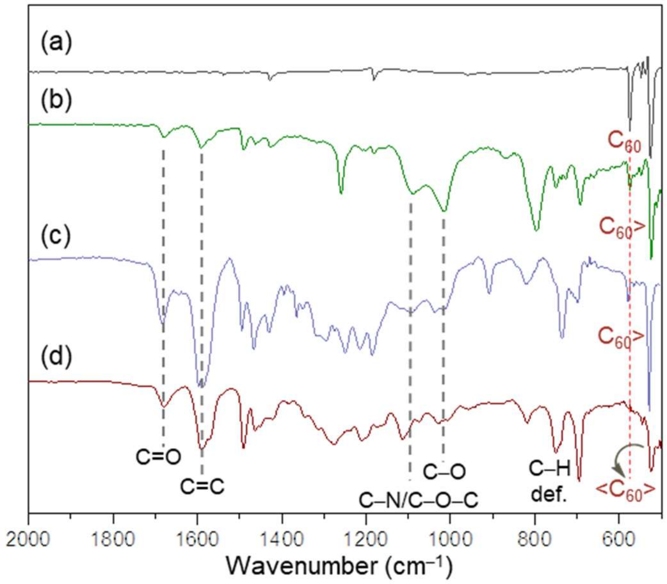

Figure S5. Infrared spectra (KBr) of (a) C_{60}, (b) C_{60}(>DPAF-C_9) (**1**-C_9), (c) tris[C_{60}(>DPAF-C_9)], and (d) tris[(DPAF-C_{2M})-C_{60}(>DPAF-C_9)] (**4**-C_{2M-9}), showing the half-cage absorption of C_{60}> and <C_{60}> at 524 cm^{-1}.

Another examples for comparison can be given by the tetraadduct C_{60}(>DPAF-C_9)$_4$. In this case, two chromatographic TLC fractions of the products were isolated to display the Infrared spectra showing no characteristic absorption band of a half-C_{60} cage at 524 cm^{-1} (Figures S6d and S6e), indicating both spheres of C_{60} were functionalized by DPAF-C_9 moieties.

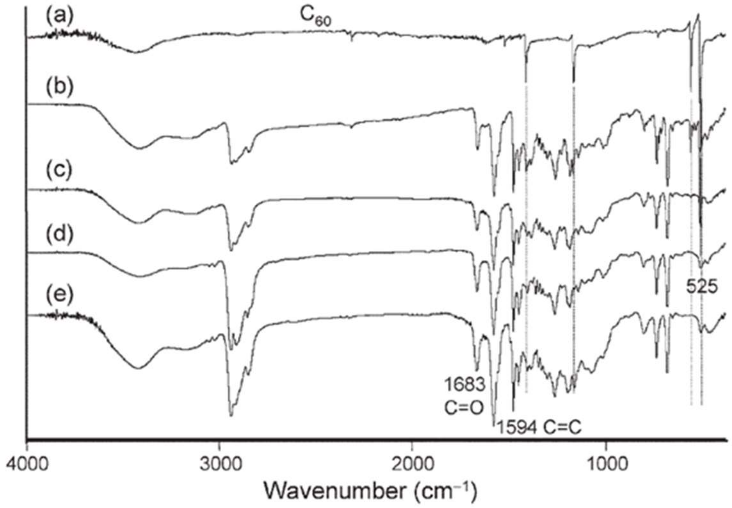

Figure S6. Infrared spectra of (a) pure C_{60}, (b) C_{60}(>DPAF-C_9) **1**, (c) C_{60}(>DPAF-C_9)$_2$, (d) the first chromatographic TLC fraction of C_{60}(>DPAF-C_9)$_4$, and (e) the second chromatographic TLC fraction of C_{60}(>DPAFC_9)$_4$ [S6].

Determination of three equivalent C_{60}(>DPAF-C_9) moieties vs. one tris(DPAF-C_{2M}) (2-C_{2M}) moiety in tris[(DPAF-C_{2M})-C_{60}(>DPAF-C_9)] (4-C_{2M-9}).

We used the proton integrations in ^{1}H NMR spectra to calculate and count for the molar quantity ratio among fluorene moieties, methoxyethyl moieties, and C_9 alkyl moieties to prove the molecular formulation of tris[(DPAF-C_{2M})–C_{60}(>DPAF-C_9)] (**4**-C_{2M-9}).

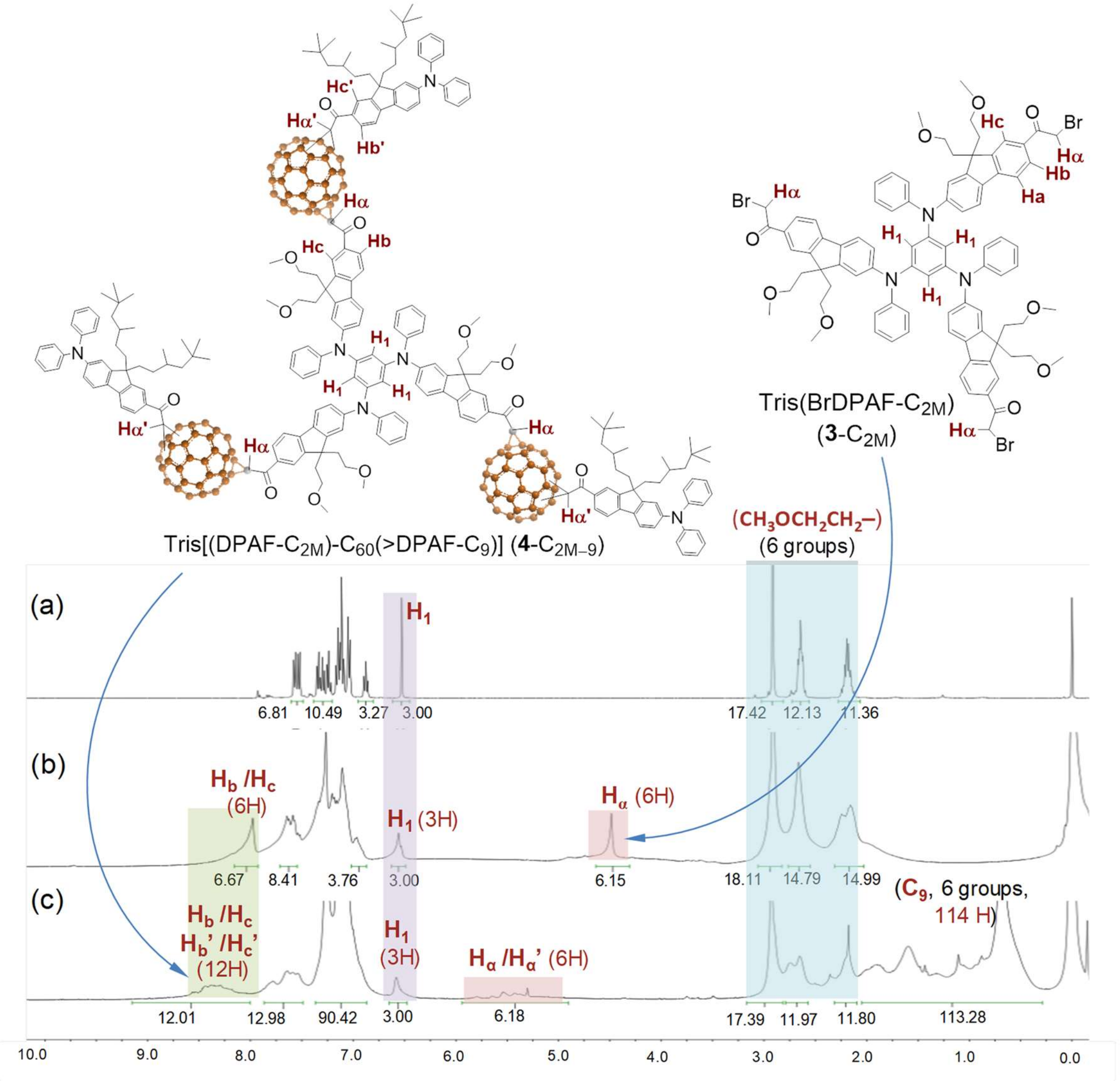

Figure S7. ^{1}H NMR spectra of (a) tris(DPAF-C_{2M}), (b) tris(BrDPAF-C_{2M}), and (c) tris[(DPAF-C_{2M})-C_{60}(>DPAF-C_9)] in $CDCl_3$, with the proton integration ratio values provided.

The peak from δ 8.12–8.65 (Figure S7c) has an integration value of 12.01 which represents the 12 fluorene protons (H_b, H_c, H_b', and H_c' of **4**-C_{2M-9}) next to keto group (3Hs for each proton group). Tris(BrDPAF-C_{2M}) (**3**-C_{2M}) contains three DPAF moieties which should have a total of 6 protons (three for each of H_b and H_c) next to the keto group in this region (Figure S7b). When three C_{60}(>DPAF-C_9) attached on tris(DPAF-C_{2M}), another 6 protons (three for each of H_b' and H_c') were added to this region to account for a total of 12 protons. This was consistent with the integration value of 12.01 measured (Figure S7c). The peak from δ 7.48–7.91 has an

integration value of 12.98 which represents the 12 fluorene protons (2Hs for each fluorene moiety). The peak from δ 7.06−7.31 has an integration value of 90.42 which represents the sum of 45 aminophenyl protons (5Hs for each of 9 aminophenyl moieties) and 12 fluorene protons (2Hs for each fluorene ring) next to nitrogen atom. However, due the overlap of these protons and the solvent residue peak of $CDCl_3$ (centered at δ 7.26), the integration value was more than the calculated value of 57. The peak at δ 6.57 has an integration value of 3.0 which represents the aromatic proton (H_1) of central phenyl ring. The peak from δ 5.25–5.78 has an integration value of 6.18 which represents the 6 α-protons (H_α and H_α') next to C_{60}> (Figure S7c). The peak from δ 2.81–3.01 (centered at 2.93) has an integration value of 17.39 which represents the 18 primary CH_3 alkyl proton of $CH_3OCH_2CH_2$– (C_{2M}) next to the oxygen atom (3Hs for each of 6 methoxy groups). The peak from δ 2.50–2.81 (centered at δ 2.65) has an integration value of 11.97 which represents the 12 methylenoxy (–OCH_2–) protons next to oxygen atom (2Hs for each of 6 C_{2M} groups). The peak from δ 2.07–2.48 (centered at δ 2.17) has an integration value of 11.80 which represents the 12 alkyl protons (–CH_2-fluorene) next to the fluorene ring (2Hs for each of 6 C_{2M} groups). The peak from δ 0.30–2.07 has an integration value of 113.28 which represents the 114 alkyl protons of 6 branched C_9 groups (19Hs for each of C_9 group). Assignments of some chemical shift values were correlated to those of C_{60}(>DPAF-C_9)

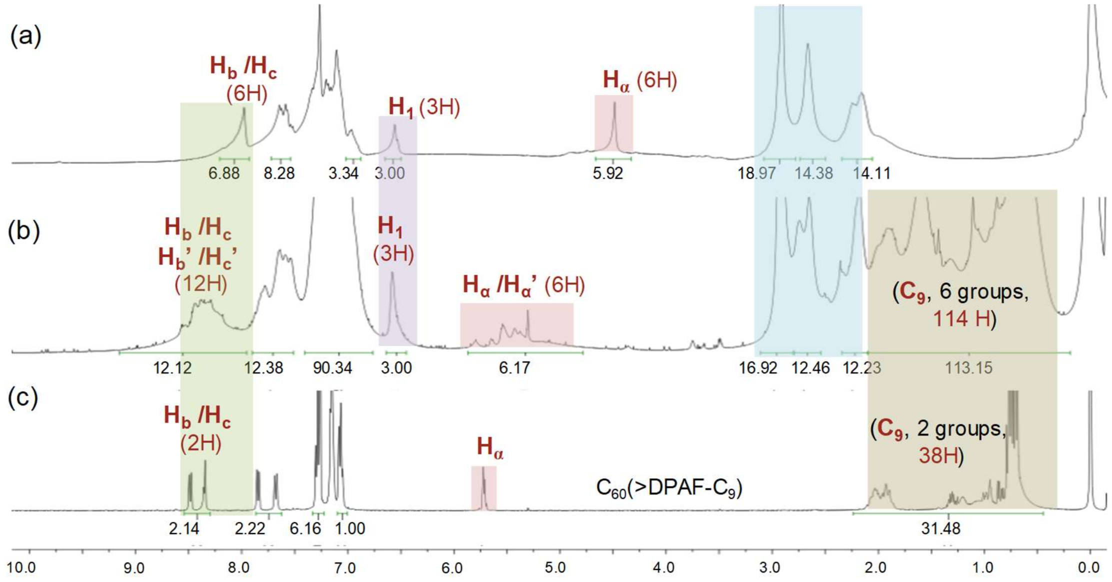

Figure S8. ^{1}H NMR spectra of (a) tris(BrDPAF-C_{2M}), (b) tris[(DPAF-C_{2M})-C_{60}(>DPAF-C_9)], and (c) C_{60}(>DPAF-C_9) (as a reference of chemical shift values) in $CDCl_3$, with the proton integration ratio values provided.

Reference

S1. Yin, H.; Wang, M.; Yu, T.; Tan, L.-S.; Chiang, L. Y. 3D-conformer of tris[60]fullerenylated *cis*-tris(diphenylaminofluorene) as photoswitchable charge-polarizer on GHz-responsive trilayered core-shell dielectric nanoparticles. *Molecules* **2018**, *23*, 1873–1889.

S2. Kang, N.-G.; Kokubo, K.; Jeon, S.; Wang, M.; Lee, C.-L.; Canteenwala, T.; Tan, L.-S.; Chiang, L. Synthesis and photoluminescent properties of geometrically hindered *cis*-tris(diphenylaminofluorene) as precursors to light-emitting devices. *Molecules* **2015**, *20*(3), 4635–4654.

S3. Lee, Y.-T.; Wang, M.; Kokubo, K.; Kang, N.-G.; Wolf, L.; Tan, L.-S.; Chen, C.-T.; Chiang, L. New 3D-stereoconfigurated *cis*-tris(fluorenylphenylamino)-benzene with large steric hindrance to minimize π–π stacking in thin-film devices. *Dyes Pigm*. **2018**, *149*, 377–386.

S4. Chiang, L. Y.; Padmawar, P. A.; Canteenwala, T.; Tan, L.-S.; He, G. S.; Kannan, R.; Vaia, R.; Lin, T.-C.; Zheng, Q.; Prasad, P. N. Synthesis of C_{60}-diphenylaminofluorene dyad with large 2PA cross-sections and efficient intramolecular two-photon energy transfer. *Chem. Comm*. **2002**, 1854–1855.

S5. Padmawar, P. A.; Canteenwala, T.; Tan, L.-S.; Chiang, L. Y. Synthesis and characterization of photoresponsive diphenylaminofluorene chromophore adducts of [60]fullerene. *J. Mater. Chem*. **2006**, *16*, 1366−1378.

S6. Elim, H. I.; Anandakathir, R.; Jakubiak, R.; Chiang, L. Y.; Ji, W.; Tan, L. S. Large concentration-dependent nonlinear optical responses of starburst diphenylaminofluorenocarbonyl methano[60]fullerene pentaads. *J. Mater. Chem*. **2007**, *17*, 1826−1838.
